# Sequential serological surveys in the early stages of the coronavirus disease epidemic: limitations and perspectives

**DOI:** 10.1590/0037-8682-0351-2020

**Published:** 2020-07-03

**Authors:** Marcelo Adriano da Cunha e Silva Vieira, Chrystiany Plácido de Brito Vieira, Amaríles de Souza Borba, Maria Clara de Carvalho Melo, Marilene de Sousa Oliveira, Rodrigo Moraes Melo, Vanessa Veloso Nunes, Wesllany Sousa Santana, Yara Amorim de Aguiar

**Affiliations:** 1 Fundação Municipal de Saúde, Diretoria de Vigilância em Saúde, Teresina, PI, Brasil.; 2 Instituto Evandro Chagas, Programa de Pós-Graduação em Virologia, Ananindeua, PA, Brasil.; 3 Ministério da Saúde, Secretaria de Vigilância em Saúde, Brasília, DF, Brasil.; 4 Universidade Federal do Piauí, Departamento de Enfermagem, Teresina, PI, Brasil.; 5 Fundação Municipal de Saúde, Centro de Operações de Emergências em Saúde Pública, Teresina, PI, Brasil.; 6 Universidade Federal do Piauí, Programa de Pós-Graduação em Saúde e Comunidade, Teresina, Piauí, Brasil.; 7 Instituto de Pesquisa Opinar, Teresina, PI, Brasil.; 8 Centro Universitário Uninovafapi, Programa de Pós-Graduação em Saúde da Família, Teresina, PI, Brasil.; 9 Fundação Oswaldo Cruz, Programa de Pós-Graduação em Medicina Tropical, Teresina, PI, Brasil.

**Keywords:** Coronavirus disease, Health surveys, Pandemic

## Abstract

**INTRODUCTION:**

Estimates of the number of individuals infected by severe acute respiratory syndrome coronavirus 2 are important for health planning and establishment of expectations regarding herd immunity.

**METHODS:**

Seven testing rounds of a serological survey were conducted at 1-week intervals between April 19 and May 31, 2020 in Teresina municipality.

**RESULTS:**

Over the 7 weeks, serological positivity increased from 0.56% (95% confidence interval [CI]: 0.18%-1.30%) to 8.33% (95% CI: 6.61%-10.33%), representing 33-53 persons infected for each reported case.

**CONCLUSIONS:**

Serological screening may be an important tool for understanding the immunity of a population and planning community interventions.

The outbreak of coronavirus disease (COVID-19) was declared a public health emergency of international concern by the World Health Organization (WHO) by the end of January 2020^1^. The first imported case of the disease in Brazil was confirmed in February 2020 in the State of São Paulo. After a month, the Brazilian Ministry of Health (MOH) recognized community transmission of severe acute respiratory syndrome coronavirus 2 (SARS-CoV-2) infection throughout the national territory. Approximately 514,000 confirmed cases of the disease were reported in Brazil as of May 31, 2020, including nearly 29,000 deaths^2^. 

According to the MOH, because of the shortage of diagnostic tests, most municipal monitoring systems have restricted testing to moderate-to-severe cases of COVID-19 or those affecting health care professionals^3^. Thus, official statistics do not include mild or asymptomatic COVID-19 cases. It is estimated that up to 85% individuals infected with SARS-CoV-2 are asymptomatic or oligosymptomatic^4^. Mild or asymptomatic infections do not impact the health care system directly; however, individuals with mild or asymptomatic infection may infect others and may possibly develop a degree of immunity to reinfection. Therefore, estimates of the total number of infected individuals in a region are important for health care planning, projection of the epidemiological curve in the short and medium term, scaling of demand for medical and hospital services, and the establishment of expectations regarding group or herd immunity^5^. 

In May 2020, Piauí occupied the 20th position in the national ranking of Brazilian States by COVID-19 incidence^2^. It is possible that Piauí was in a “delayed epidemic moment” compared to other States of the federation. Data from the National Civil Aviation Agency indicate that the State capital airport (Teresina) has the second-lowest passenger flow in the country among the State capitals, just ahead of Aracajú airport in Sergipe State, which may in part justify this temporal difference^6^. 

Given the possibility of the under-reporting of COVID-19 cases and the impossibility of accounting for asymptomatic or oligosymptomatic SARS-CoV-2 infections through official records, the Municipal Health Foundation of Teresina decided to perform sequential serological surveys. This was intended to analyze the dynamics and dimension of the epidemic in the city, plan health care interventions, and project demand for hospital care. 

Serologic surveys were performed in seven testing rounds conducted at 1-week intervals between April 19 and May 31, 2020. Each round comprised testing of 900 individuals under probability sampling; they were stratified by sex, age, and geographical distribution in the city. These data were extracted from the household registry of 78 basic health units in the urban area of the capital. The study was approved by the National Research Ethics Committee, (registry no. 123.444). After signing an informed consent form, the individuals were tested using a rapid COVID-19 test (Guangzhou Wondfo Biotech Co, China), which was performed according to the manufacturer’s instructions, regardless of the presence or duration of symptoms. The diagnostic kit provides a qualitative result (positive or negative); however, it does not differentiate immunoglobulin (Ig) M and IgG antibodies. According to the manufacturer, it has a sensitivity of 86% and a specificity of 99% only if it is performed at least 8 days after the infection and has been certified by the National Institute for Quality Control in Health. 

Figure 1 shows the timeline progression of the positivity rate in the population sampled, with 95% confidence intervals (CIs) and a significance of 5%. From the third survey, the central estimate of positivity was higher than the false-positive rate expected for the test (1-specificity). In the fifth survey, it possible to certify a significant increase in the seroprevalence in the population sampled (*P* < 0.01), with a positive result detected in 3.78% (95% CI: 2.63%-5.24%) of the individuals tested. 

**FIGURE 1:**
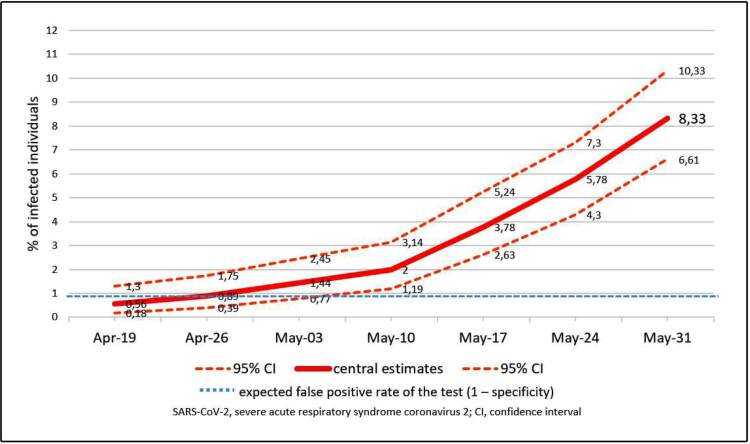
Percentage of serological positivity for severe acute respiratory syndrome coronavirus in the Teresina population at seven successive timepoints

The surveys reflected that SARS-CoV-2 infections are typically mild or asymptomatic and that data on reported cases does not represent the risk of infection. Up to 8 days before the completion of the seventh survey, the municipality of Teresina recorded 1,699 confirmed COVID-19 cases, including 51 deaths^7^. Given an estimated population of 864,845 inhabitants according to the Brazilian Institute of Geography and Statistics (IBGE), the seroprevalence in the seventh survey (8.33%, 95% CI: 6.61%-10.33%) suggests that 57,166-89,338 individuals had been infected in the capital. This represents 33-53 persons infected for each reported case. Therefore, the fatality rate of 3% calculated using the number of confirmed cases is overestimated and may reflect only the fatality rate among moderate-to-severe cases. When considering the estimates for the total number of infected individuals, the infection fatality rate varied between 0.06% and 0.09%.

The results of the serological surveys performed in Teresina permit the estimation of the progression of the total number of infected individuals over the 7 weeks studied. However, future projections of results are limited, given the low positivity in the sample, the overlapping of CIs that are attributable to the percentage of positive tests, and the expected limit for false-positive results of the diagnostic tests. Therefore, estimates of the CIs of the reproduction number calculated between each round of the study were very imprecise. However, the sensitivity of a test is generally assessed by its’ manufacturer by testing samples from more severe cases with a diagnosis confirmed by methods considered a reference standard. In these patients, the immune response can be more exuberant, which can lead to an overestimation of the test’s sensitivity. In addition, the tests used in the present study detect total antibodies against SARS-CoV-2. The use of diagnostic tests capable of discriminating IgG from IgM positivity would more reliably reflect the emergence of the actual recent infections at every stage of the study, although the dynamics of serological response to SARS-CoV-2 infection remains obscure. 

Similar initiatives with State coverage have been implemented by researchers from the Federal University of Pelotas (UFPel), Rio Grande do Sul State University, and the Federal University of Piauí State, in partnership with the respective State Public Health Departments. However, the positivity rates revealed in the initial stages have not exceeded the percentage of false-positive results estimated by the manufacturer of the diagnostic tests employed (1%)^8^ ^,^ ^9^. A study conducted in the municipality of São Paulo detected a serologic positivity for SARS-CoV-2 in 5% of the individuals tested, yet the sampling did not cover all neighborhoods of the city and was concentrated in the neighborhoods with the highest number of COVID-19 cases^10^. In May 2020, a serological survey involving several Brazilian cities was initiated by he UFPel, which was endorsed by the MOH with the participation of IBGE^11^. In June 2020, a study performed in the State of Rio de Janeiro indicated that 28% blood donors had already produced antibodies against COVID-19^12^. The results of the first nationwide seroprevalence study in Spain showed that only 5% of the population had antibodies against SARS-CoV-2 based on a rapid antibody test^13^.

Serological screening is an important tool that is used to understand the immunity of a population and distinguish individuals who are at lower risk of becoming ill. Nevertheless, even though the serologic positivity for SARS-CoV-2 reflects the detection of serum antibodies produced in response to infection, the WHO recommends caution in drawing conclusions regarding temporary or permanent immunity against COVID-19 in individuals with positive tests^14^. In the absence of a short-term expectation for the large-scale implementation of vaccines against COVID-19 and considering the expected impact of the pandemic on the population’s health, knowledge of the immune status of the inhabitants of a region and the proportion of infected people who will require hospital equipment is essential for public health managers to plan appropriate interventions. 
